# Japanese Encephalitis Virus and *Schizophyllum commune* Co-Infection in a Harbor Seal in Japan

**DOI:** 10.3390/vetsci11050215

**Published:** 2024-05-13

**Authors:** Marina Fujii, Soma Ito, Etsuko Katsumata, James K. Chambers, Hiromichi Matsugo, Akiko Takenaka-Uema, Shin Murakami, Kazuyuki Uchida, Taisuke Horimoto

**Affiliations:** 1 Laboratory of Veterinary Microbiology, Graduate School of Agricultural and Life Sciences, University of Tokyo, Tokyo 113-8657, Japan; marina.fujii@colostate.edu (M.F.); matsugo.hiromichi.2w@kyoto-u.ac.jp (H.M.); atakiko@g.ecc.u-tokyo.ac.jp (A.T.-U.); shin-murakami@g.ecc.u-tokyo.ac.jp (S.M.); 2 Laboratory of Veterinary Pathology, Graduate School of Agricultural and Life Sciences, University of Tokyo, Tokyo 113-8657, Japan; s-ito@nibs.or.jp (S.I.); achamber@g.ecc.u-tokyo.ac.jp (J.K.C.); 3 Kamogawa Sea World, Kamogawa, Chiba 296-0041, Japan; etsuko_katsumata@granvista.co.jp

**Keywords:** *Phoca vitulina*, Japanese encephalitis virus, *Schizophyllum commune*, genotype, pathogenicity

## Abstract

**Simple Summary:**

Japanese encephalitis virus (JEV) infections in seals are limited, with only two cases reported. Here, we report a case of meningoencephalitis and bronchopneumonia caused by the co-infection of JEV and *Schizophyllum commune* in a dead seal (*Phoca vitulina*) housed in an aquarium in Japan. The JEV isolate from the seal was classified as genotype GIb based on *E* gene sequences, which included recent Japanese human and mosquito isolates.

**Abstract:**

The Japanese encephalitis virus (JEV), a mosquito-borne flavivirus, has a wide host range, extending from pigs and ardeid birds to opportunistic dead-end hosts, such as humans and horses. However, JEV encephalitis infections in aquatic mammals are rare, with only two cases in seals reported to date. Here, we report a lethal case of JEV and *Schizophyllum commune* co-infection in an aquarium-housed harbor seal in Japan. We isolated JEV from the brain of the dead seal and characterized its phylogeny and pathogenicity in mice. The virus isolate from the seal was classified as genotype GIb, which aligns with recent Japanese human and mosquito isolates as well as other seal viruses detected in China and Korea, and does not exhibit a unique sequence trait distinct from that of human and mosquito strains. We demonstrated that the seal isolate is pathogenic to mice and causes neuronal symptoms. These data suggest that seals should be considered a susceptible dead-end host for circulating JEV in natural settings.

## 1. Introduction

Japanese encephalitis (JE) is an acute, severe viral encephalitis associated with high mortality and morbidity rates. Annually, over 60,000 JE cases are reported across Asian countries, including the Southeast Asia and West Pacific regions [1]. Although >99% of human infections are asymptomatic, the mortality rate reaches approximately 30% when symptoms manifest [2,3,4]. 

The etiological agent of JE, the Japanese encephalitis virus (JEV), is a member of the family *Flaviviridae* and the genus *Orthoflavivirus*. JEV is maintained throughout the transmission cycle between the amplifying vertebrate hosts and *Culex* spp. mosquitoes. Pigs and ardeid birds develop high-titer viremia and serve as the amplifying hosts of JEV [5,6,7,8,9]. Although JEV does not cause encephalitis in adult pigs, it triggers reproductive disorders such as stillbirth, abortion, and congenital malformations in the fetus [10,11], causing significant economic losses to the livestock industry. Considering there is no specific treatment for JE, vaccination is the only way to effectively control JEV infection in both humans and animals [12,13].

The serological evidence of JEV infection has been reported in a range of domestic and wildlife species. Although most JEV infections are subclinical, horses often develop fatal encephalitis [14,15]. Similarly, JEV infection in cattle is common in endemic regions, and infection during pregnancy is frequently associated with miscarriage [16,17]. Horses and cattle are considered dead-end hosts because of their low-titer viremia [18,19]. Additionally, JEV genome or JEV-specific antibodies have been detected in various forms of terrestrial wildlife, including bats, monkeys (*Macara fuscata*), wild boars (*Sus scrofa*), feral raccoons (*Procyon lotor*), and raccoon dogs (*Nyctereutes procyonoides*) [20,21,22,23]. The pathophysiology of JEV infection in wild animals and their ecological role remain largely unknown. In aquatic animals, JEV infection is unlikely to occur in their natural habitats because of limited exposure to mosquito vectors. However, they may become susceptible to JEV in artificial settings where they are exposed to mosquitoes. Notably, two JE cases involving three animals were reported in aquarium-kept speckled seals (*Pusa hispida:* former *Phoca hispida*) in China [24] and a spotted seal (*Phoca largha*) in the Republic of Korea [25] in 2017. 

*Schizophyllum commune* is a common basidiomycete fungus found on all continents except Antarctica. It typically grows on decaying vegetation such as rotten wood. Although rare, it occasionally causes diseases in humans. As of 2013, 71 cases of the disease have been reported globally. However, the actual number of cases may be considerably higher because it is often misdiagnosed as aspergillosis. Human infection with *S. commune* often manifests as bronchopulmonary diseases and sinusitis. Recently, the involvement of *S. commune* in ophthalmic disease has also been indicated [26,27]. Reports of *S. commune* infection in animals are even rarer, with only four cases documented in dogs [28,29,30,31] and one case in seals [32] to date. The pathological manifestations in dogs differ from those in humans, which include three cases of osteomyelitis and one case of subcutaneous granuloma. Notably, a harbor seal (*Phoca vitulina*) in a zoo in Japan died after presenting with corneal opacity and labored breathing. Severe necrotizing and granulomatous inflammation with fungal organisms were observed in the eyes, lungs, heart, and lymph nodes [32].

In the present study, we report a lethal case of an aquarium-kept harbor seal co-infected with JEV and S. commune. The aim of our study is a post-mortem examination to discover the cause of death of this animal.

## 2. Materials and Methods

### 2.1. Pathological Examination

For the necropsy, tissue samples were collected, fixed in 10% phosphate-buffered formalin, and embedded in paraffin. Formalin-fixed paraffin-embedded (FFPE) tissues were sliced into 4 μm thick sections and stained with hematoxylin and eosin. Selected tissue sections of the lungs and tracheobronchial lymph nodes were stained with Periodic acid–Schiff (PAS) and Grocott’s methenamine silver (GMS) stains. An immunohistochemistry (IHC) of the brain tissue sections was performed using the primary antibodies listed in Table 1. Deparaffinized tissue sections were immersed in 10% hydrogen peroxide (H_2_O_2_) in methanol at 23 °C for 5 min. For the JEV envelope protein, antigen retrieval was performed via an autoclave pretreatment conducted at 121 °C for 10 min using a Target Retrieval Solution, at a pH of 9.0 (Dako, Santa Clara, CA, USA). Following this, the tissue sections were incubated in 8% skim milk in Tris-buffered saline at 37 °C for 40 min to prevent non-specific reactions. Each tissue section was subsequently incubated with the primary antibody at 4 °C overnight. An anti-rabbit IgG polymer labeled with horseradish peroxidase (Envision, Agilent Technology, Santa Clara, CA, USA) was applied at 37 °C for 40 min and tissue sections were then rinsed with Tris-buffered saline. The reactions were visualized with 0.05% 3-3′-diaminobenzidine containing 0.03% H_2_O_2_ in Tris-hydrochloric acid buffer, followed by a counterstain with Mayer’s hematoxylin. For IHC of JEV, a brain tissue section from a JEV-infected pig was used as the positive control.

### 2.2. Pathogen Detection

Genomic DNA was extracted from FFPE tissue samples of the lung and tracheobronchial lymph node using the QIAamp DNA FFPE Tissue Kit (Qiagen, Hilden, Germany), and a panfungal polymerase chain reaction (PCR) was performed using the following primers: ITS3-F (5′-GCATCGATGAAGAACGCAGC-3′) and ITS4-R (5′-TCCTCCGCTTATTGATATGC-3′) [33]. Total RNA was extracted from frozen cerebral tissue samples using an RNeasy Plus Mini Kit (Qiagen). cDNA was synthesized from the cerebral RNA using a PrimeScript RT-PCR Kit (Takara Bio, Shiga, Japan). RT-PCR for the detection of mosquito-borne flaviviruses was performed using the following primers: FLAVI-1 (5′-AATGTACGCTGATGACACAGCTGGCTGGGACAC-3′) and FLAVI-2 (5′-TCCAGACCTTCAGCATGTCTTCTGTTGTCATCCA-3′) [34]. For both PCRs, the total reaction volume used was 25 μL containing 12.5 µL of KOD One PCR Master Mix-Blue- (Toyobo, Osaka, Japan), 0.75 µL of forward primer (10 µM), and 0.75 µL of reverse primer (10 µM). Template DNA (30–40 ng) was added to each PCR. Distilled water was used as a negative control instead of template DNA. DNA fragments were amplified according to PCR cycle conditions described in previous studies [33,34]. The amplified products were purified using a High Pure PCR Product Purification Kit (Roche Diagnostics, Mannheim, Germany), following agarose gel electrophoresis, and subjected to Sanger sequencing. The obtained sequences were analyzed using the Basic Local Alignment Search Tool (BLAST) from the National Center for Biotechnology Information (NCBI; https://blast.ncbi.nlm.nih.gov/Blast.cgi) (accessed on 1 March 2024) [35].

### 2.3. Cells and Viruses

Baby hamster kidney BHK21 (American Type Culture Collection [ATCC], Manassas, VA, USA, CCL-10), African green monkey kidney Vero (ATCC, CCL-81), hamster lung HmLu-1 (National Institute of Animal Health, Japan, Ibaraki, Japan), and *Aedes albopictus* C6/36 (ATCC, CRL-1660) cell lines were used. Vertebrate-derived cells were propagated in Dulbecco’s modified essential medium (DMEM; Fujifilm Wako, Osaka, Japan), supplemented with 5% fetal bovine serum (FBS) and kept at 37 °C in a 5% CO_2_ incubator. C6/36 cells were propagated in a modified essential medium (MEM; Life Technologies/Gibco, Paisley, UK), supplemented with 5% FBS and non-essential amino acid solution (Fujifilm Wako), and kept at 28°C. Two live attenuated vaccine strains for swine, namely the m strain (Kyoto Biken Laboratories, Kyoto, Japan) [12] and the at strain (Nisseiken, Tokyo, Japan) [36], were procured, underwent a single passage in BHK21 cells, and subsequently stored at −80 °C prior to use.

### 2.4. Virus Isolation

Brain sample from the dead harbor seal, frozen at −80 °C, was thawed and finely chopped into small pieces. The pieces were then transferred into a 2 mL tube along with 5 mm stainless steel beads (Qiagen). Subsequently, DMEM with 1% FBS was added to the tube to obtain a final brain homogenate concentration of 10%. The tube was then processed using a Tissue Lyser II (Qiagen). The resultant homogenate was filtered through a 450 nm membrane filter and diluted 1:100 with DMEM containing 1% FBS. BHK21 cells (70% confluent) in a 6-well plate were inoculated with 200 µL of the diluted homogenate and incubated for 60 min. The cells were then washed once with DMEM containing 1% FBS, overlaid with 2 mL of the same medium, and incubated until cytopathic effects (CPEs) were observed. The culture supernatant (designated P1) was harvested on day 4 when the CPEs became clear. The virus was cloned via two rounds of limiting dilution in BHK21 cells (P2 and P3). Subsequently, the viral stock was then divided into aliquots (P4).

### 2.5. Plaque Formation

BHK21, Vero, and HmLu-1 cell monolayers were tested for plaque formation. The cells were transferred to 6-well plates and incubated with 200 µL of viral inoculum for 1 h at 37 °C. After removing the inoculum, the cells were overlaid with DMEM supplemented with 2% FBS and 0.8% SeaKem GTG agarose (Lonza, Chiba, Japan). Subsequently, the cells were incubated at 37 °C for 3–7 days until the plaque grew to a countable size. Cells were fixed in formalin and stained with crystal violet for plaque visualization.

### 2.6. Mouse Study

Viral titers (plaque forming units, PFU) were determined using plaque assays in BHK21 cells. Four-week-old female ICR mice (Japan SLC, Shizuoka, Japan) were intraperitoneally injected with 1.0 (*n* = 2) or 10^3^ pfu (*n* = 2) of the seal isolate (JEV/Seal/UT1/2020) or 10^4^ PFU of live vaccine m and at strains (each *n* = 4). Following injection, the body weights of the mice were recorded, and the mice were examined for any signs of disease daily for 14 days. The mice were euthanized if their body weight decreased to less than or equal to 80% of their initial weight. In another set of experiments, mice (*n* = 3) were intramuscularly injected with various titers of JEV/Seal/UT1/2020, and the viral titers in brain samples were subsequently measured in euthanized mice. Furthermore, the brains were examined histologically according to the procedure described above.

### 2.7. Sequencing

Viral particles were purified from the supernatants of infected cell cultures via ultracentrifugation at 10,986× *g* for 3 h using a P32ST rotor (Himac, Tokyo, Japan) with a 20% sucrose cushion. Viral RNA was extracted from the purified virus using ISOGEN-LS (Nippon Gene, Tokyo, Japan), following the manufacturer's instructions. The whole-genome sequence of the seal isolate was determined using next-generation sequencing and deposited in GenBank under accession number LC687612. For live vaccine strains, RNAs were reverse-transcribed using ReverTra Ace (Toyobo) with random 6-mers. Two overlapping segments comprising the envelope (*E*) gene were PCR-amplified with KOD FX Neo (Toyobo). The amplified products were purified using agarose gel electrophoresis and subjected to Sanger sequencing. We deposited the *E* gene sequences for the at and m strains to GenBank under the accession numbers LC687613 and LC701057, respectively. The primer sequences for PCR amplification are available upon request.

### 2.8. Phylogenetic Analysis

The phylogenetic tree of *E* gene sequences of JEV strains since 2015 was constructed using MEGA version 7 [37] employing maximum likelihood analysis with 1000 bootstraps. Vaccine and seal strains were incorporated into the dataset. Additionally, one representative strain of a known phylogeny was chosen to ensure the inclusion of every genotype or sub-genotype.

## 3. Results

### 3.1. The Case

A wild harbor seal originally captured in the sea area of Hokkaido, northern Japan, in 2016 was later transferred to an aquarium in Chiba, eastern Japan. In April 2019, the animal developed anorexia. Blood tests revealed a high white blood cell (WBC) count of 43,500/μL, a β-D-glucan value of 45.6 pg/mL, and an endotoxin value of 3.7 pg/mL, indicating a potential fungal and/or bacterial infection. Following treatment with the antifungal drug itraconazole, the seal’s appetite improved. Two weeks after the recovery in September 2019, the animal developed a fever (38.8 °C) with an elevated WBC count of 23,700/μL. Another antifungal drug, voriconazole, appeared to be effective and was administered until 15 September 2020. However, on 1 October the seal once again showed a high fever (39.1 °C) and an elevated β-D-glucan value of 112 pg/mL. Although a fungal infection was suspected, the Aspergillus antigen test result was negative, suggesting the involvement of another fungal pathogen. Voriconazole treatment was resumed, but unfortunately, the animal died on 7 October. No apparent neuronal symptoms were observed before death. No other animals in the same pool exhibited any signs of illness.

### 3.2. Pathological Findings

**Figure 1:**
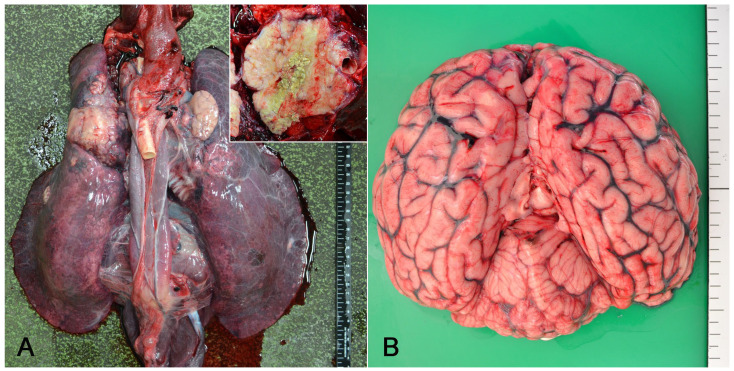
Gross findings of a harbor seal co-infected with *Schizophyllum commune* and Japanese encephalitis virus. (**A**) The lungs exhibited multiple whitish nodules, and the tracheobronchial lymph nodes appeared enlarged. Upon sectioning, a yellowish-white material was observed at the center of the nodule (inset). (**B**) The congestion of meningeal vessels was observed in the brain.

Upon necropsy, nodular lesions were observed in the lungs: a single large nodule (10 cm in diameter) in the left cranial lobe, a single large nodule (6 cm in diameter) in the right cranial lobe, and multiple small nodules (<3 cm in diameter) in both the caudal lobes. The nodules were firm and well demarcated (Figure 1A). Yellowish-white material was found in the center of the nodules upon sectioning. In addition, tracheobronchial lymph nodes were enlarged (6.5 × 3.5 cm), and the cut surface showed multiple nodules similar to those in the lung. The meningeal blood vessels were severely congested (Figure 1B).

**Figure 2:**
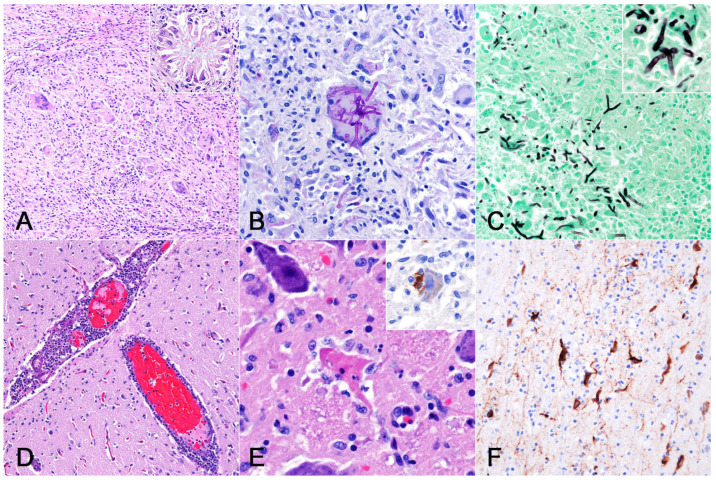
Histological findings of a harbor seal co-infected with *Schizophyllum commune* and Japanese encephalitis virus (JEV). (**A**) Lung. Granulomatous inflammation with fibrosis. Macrophages, including multinucleated giant cells, surrounded fungal organisms (inset). Stained with hematoxylin and eosin (HE). (**B**) Lung. Fungi phagocytosed by multinucleate giant cells had septate hyphae with Y-shaped branching. Stained with PAS. (**C**) Tracheobronchial lymph node. Fungi were stained black and displayed morphological features similar to those observed in the lung (inset). Stained with Grocott’s methenamine silver. (**D**) Cerebrum. Congestion and inflammatory cell infiltration in the meninges and perivascular cuffing in the cortex. Stained with HE. (**E**) Medulla oblongata. A necrotic neuron surrounded by phagocytes including neutrophils (i.e., neuronophagia) in the magnocellular reticular nucleus. Some phagocytes were immunopositive for myeloperoxidase (inset; dorsal motor nucleus of the vagus nerve). Stained with HE. (**F**) Cerebrum. JEV antigens detected in the cytoplasm of cortical neurons. Note that the antigens were distributed along the neurites. Staining was observed using immunohistochemistry.

Histologically, the pulmonary nodules were characterized by multifocal granulomatous inflammation with fibrosis, leading to the replacement of the alveolar structures (Figure 2A). Within these nodules, numerous epithelioid macrophages, including multinucleated giant cells, infiltrated and frequently contained phagocytosed fungi. Staining with PAS and GMS clarified the characteristic morphology of the fungi: filamentous septate hyphae (3–5 μm wide) with Y-shaped branching (Figure 2B). These findings were also observed in tracheobronchial lymph nodes (Figure 2C). Diffuse meningeal congestion and perivascular cuffing were observed in the cerebrum (Figure 2D). Inflammatory cells include numerous mononuclear cells and small numbers of neutrophils and eosinophils. Thrombus formation was observed in several blood vessels. Glial nodules and neuronal necrosis were observed in the cortex. Necrotic neurons have eosinophilic cytoplasm and nuclear debris, often surrounded by phagocytes (i.e., neuronophagia). The phagocytes consisted mainly of mononuclear cells, occasionally accompanied by neutrophils immunopositive for myeloperoxidase (Figure 2E). These findings were also observed in the olfactory bulb, thalamus, midbrain, and medulla oblongata. Upon the IHC, the JEV envelope protein was detected within the cytoplasm of neurons of the olfactory bulb, piriform cortex, entorhinal cortex, nucleus of the thalamus, and midbrain (Figure 2F).

### 3.3. Molecular Identification of Pathogens

The panfungal PCR successfully amplified a DNA fragment of approximately 400 bp from lung and tracheobronchial lymph node samples. The sequenced PCR products exhibited >99% homology with *S. commune* reference sequence (GenBank accession number: MT466518.1). The RT-PCR for the detection of mosquito-borne flaviviruses amplified a DNA fragment of approximately 820 bp from the cerebrum sample, and the sequenced PCR products exhibited >99% homology with the JEV reference sequence (GenBank accession number: MK095782.1). 

### 3.4. Virus Isolation

The cell cultures were inoculated with brain homogenate samples to isolate viruses. In BHK21 cells, CPE-containing round cells were observed locally on day 2 post-inoculation, and by day 4, an expansion was noted. In Vero cells, CPE was observed as cell detachment on days 6–7. In contrast, no apparent CPE was observed in C6/36 cells until day 7, although the virus was detected through the appearance of CPE upon the inoculation of the supernatant into BHK21 cells. These findings revealed the successful isolation of seal-origin JEV. We collected the supernatant of the infected BHK21 cells and designated the strain as JEV/seal/UT1/2020. 

### 3.5. Sequence Analysis

**Figure 3:**
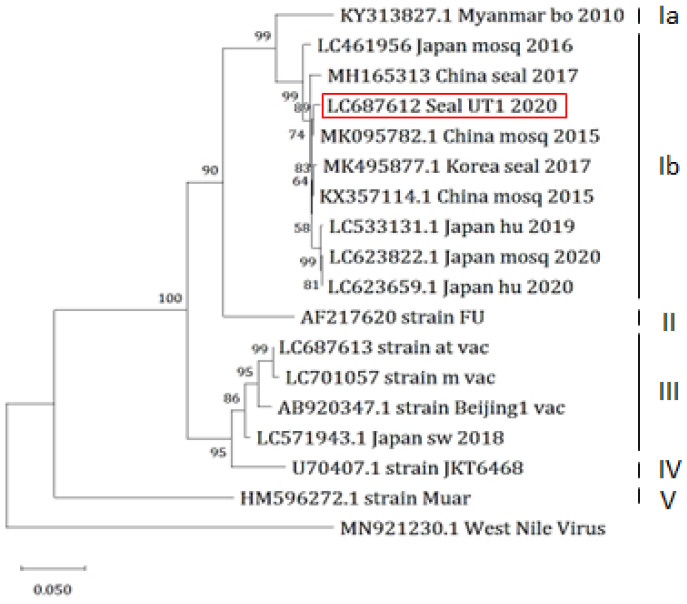
Phylogenetic tree based on E gene sequence. Genotypes (la, lb, II, III, IV, and V) are represented on the right. The scale bar refers to the phylogenetic distance of 0.05 nucleotide substitutions per site. The West Nile virus is adopted as an outgroup. The seal virus JEV/seal/UT1/2020 isolated in this study is shown within the red square. bo: bovine; mosq: mosquito; hu: human; sw: swine; vac: vaccine.

We determined the *E* gene sequence of JEV/seal/UT1/2020 and conducted a phylogenetic analysis. The analysis showed that the isolate belonged to the genotype GIb, consistent with the most recent Japanese isolates (Figure 3). A BLAST search identified strains ZJ-YW-11-15 [38] and LYG-2 [39] as the closest match, sharing a 99.3% identity, both isolated from *Culex tritaeniorhynchus* in China in 2015 and 2016. Moreover, two seal isolates in China and Korea also belonged to the genotype GIb, whereas the two live vaccine m and at strains and one inactivated vaccine seed Beijing-1 strain used in Japan were classified as genotype III. A previous study indicated that eight amino acid residues of the E protein—107L, 138E, 176I, 177T, 244E, 264Q, 279K, 315A, and 439K—are linked to viral neurovirulence, of which 138E is the most critical residue [40]. All these eight amino acids were conserved in JEV/seal/UT1/2020, whereas live vaccine strains possessed possible attenuated mutations at E138K and I176T, suggesting that the virus obtained from the seal might exhibit a virulent phenotype. 

### 3.6. Mouse Study

**Figure 4:**
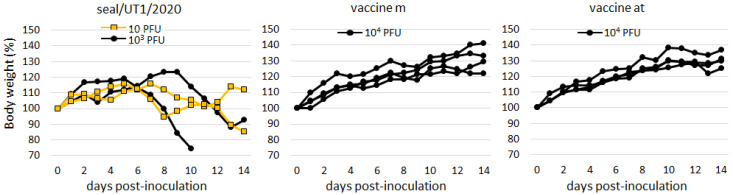
Pathogenicity of the seal virus in mice. Body weights were monitored during a two-week period following intramuscular inoculation with JEV/seal/UT1/2020 and live vaccine strains (m and at) and represented as change percentages compared with initial body weights (day 0). For the seal virus, two different titers (10 and 10^3^ PFU) were used for inoculation. For the vaccine strains, a viral titer of 10^4^ PFU was used.

Mouse experiments were performed to assess the pathogenicity of JEV/seal/UT1/2020. All four mice injected with the seal virus showed a significant reduction in body weight compared to those injected with the vaccine strains (Figure 4). Sick mice infected with the seal virus presented with symptoms indicative of neuronal disorders, such as dullness, abnormal posture, and excessive grooming. In addition, considerable titers of viruses (1.6 × 10^4^ − 6.6 × 10^6^ PFU/g) were recovered from the cerebrum samples of all three mice infected with the seal virus. The viruses were also recovered from the cerebellum (2.0 × 10^2^ PFU/g) and medulla oblongata/pons (1.6 × 10^4^ PFU/g) samples of an infected mouse. 

**Figure 5:**
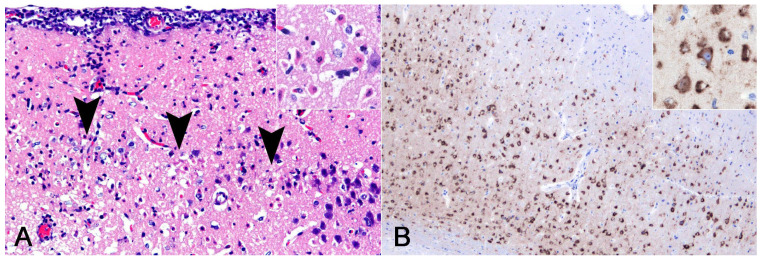
Histological findings of mice inoculated with JEV/seal/UT1/2020. (**A**) Cerebrum. Loss of neurons due to neuronal necrosis in the external pyramidal layer (arrowheads). Necrotic neurons were eosinophilic and contained nuclear debris (inset; higher magnification). Stained with hematoxylin and eosin. (**B**) Cerebrum. Japanese encephalitis virus antigens were diffusely distributed throughout the cortex. Strong immunopositivity was observed in the cytoplasm of neurons (inset; higher magnification). Staining was observed using immunohistochemistry.

The histological examination of the mice inoculated with JEV/seal/UT1/2020 revealed characteristic findings consistent with those of brain lesions in seals. Extensive neuronal loss was observed in the external pyramidal layer of the cerebral cortex (Figure 5A). The residual neurons had eosinophilic cytoplasm and nuclear debris, indicating neuronal necrosis. JEV antigens were detected in cortical neurons via IHC (Figure 5B).

## 4. Discussion

To our knowledge, this is the first reported clinical case of co-infection with JEV and *S. commune* in a harbor seal leading to complex disease outcomes, including respiratory and systemic symptoms, and eventually resulting in death. In terms of *S. commune* infection in seals, this is the second report of a lethal case; notably, the first case occurred in Tokyo, Japan [32]. The abundance of *S. commune* in the environment and the absence of the disease in other animals in the same cage suggested that the infection was opportunistic, probably due to the animal’s compromised immune status. As for JEV, there have been two reports of infection in seals, where viruses were isolated from the brains of dead animals in China [24] and Korea [25], confirming that seals are susceptible hosts for JEV infection. Interestingly, the seal in Korea was co-infected with *Dirofilaria immitis*. These findings suggest that the seal is an intrinsic dead-end host for JEV, although disease progression may vary depending on existing risk factors, such as immune status, which could be compromised by co-infections with other pathogens.

In this case, the mode of JEV transmission to the seal was undetermined. Generally, *Culex* mosquitoes move within a range of 250 m to 1 km [41,42], which can be extended to 8.4 km with the assistance of wind [43]. However, there was no pig farm within the 10 km radius of the aquarium. The surrounding area of the aquarium comprises a river, rice fields, and forests inhabited by wading birds and wild boars, suggesting the potential role of wild animals and vector mosquitoes in the JEV transmission to the seal that was occasionally housed in the open pool of the aquarium. To address this possibility, a surveillance study around the aquarium is warranted. 

Among the three mammalian cell cultures tested, BHK21 cells were the most suitable for the isolation and growth of JEV/seal/UT1/2020. In BHK21 cells, the virus formed visible plaques after day 2, with the plaques becoming countable at 2–3 mm in diameter by day 3 post-infection. In contrast, in HmLu-1 cells, clear plaques of 1–2 mm were observed by day 4. In Vero cells, the plaques formed were less distinct compared to those in BHK21 or HmLu-1 cells in terms of color contrast and the sharpness of the edges. In contrast, the two live vaccine strains formed clear plaques 1–2 mm in diameter on days 7–8 in Vero cells, whereas their plaques on BHK21 cells were distorted in shape with blurred edges, albeit still countable. These findings suggest that BHK21 cells may serve as valuable tools for detecting and isolating JEVs.

JEVs are phylogenetically classified into five genotypes, GI to GV, based on their *E* gene nucleotide sequences [44]. Southeast Asia is the only region where all five genotypes have been found and is considered the original epicenter of JEV [45]. GIII was the dominant lineage in temperate and subtropical Asia until the 1990s and 2000s when GI gradually replaced it [46,47,48,49]. GI is further divided into two sub-genotypes: GIa and GIb. The geographical distribution of GIa is limited to mainland Southeast Asia, Tibet, and Australia, while GIb exhibits a broader geographical distribution across temperate Asia [50,51]. Our seal isolate, JEV/seal/UT1/2020, belongs to genotype GIb, which includes the other two seal strains as well as recent human and mosquito viruses in Japan. The transmissibility of JEV genotypes other than GIb to seals is unknown because there is no unique amino acid substitution in the E protein shared among the three seal strains.

All vaccine strains used in Japan, including the two live vaccines, m and at strains for pigs, and the inactivated vaccine Beijing-1 strain for humans, pigs, and horses, were classified as genotype III because these vaccines were developed when GIII was the dominant lineage in Japan. Although JEVs are known to exist as a single serotype, previous studies indicated that cross-protective immunity is only partial between genotypes GI and GIII [52,53]. Therefore, the development of an alternative vaccine using a recent GIb strain is expected to provide better protection against JEV infection in humans and animals, including seals, in Japan. 

## 5. Conclusions

Here, we report a lethal co-infection of *S. commune* and JEV in a harbor seal. Granulomatous pneumonia, lymphadenitis caused by *S. commune* infection, and meningoencephalitis caused by JEV infection were confirmed. Although seals are not well recognized as being susceptible to JEV infection, this is the third case of JEV infection in seals, supporting the notion that seals are dead-end hosts for JEV infection and that vaccinating seals against this infection may be warranted for zoos and aquariums. The isolated JEV strain belonged to the genotype GIb, consistent with recent isolates from Japan. The currently available JEV vaccines were developed before the genotype shift that occurred during the 1990s; therefore, they are exclusively effective against genotype GIII. Consequently, it is necessary to re-examine the efficacy of the JEV vaccine against circulating strains. 

## Figures and Tables

**Table 1:** Primary antibodies used for immunohistochemistry.

Antigen	Type	Dilution	Antigen Retrieval	Catalog No.	Source
JEV envelope protein	Rabbit, polyclonal	1:200	Heat, pH 9.0	GTX125867	GeneTex, Irvine, CA, USA
Myeloperoxidase	Rabbit, polyclonal	1:400	None	A0398	Dako, Santa Clara, CA, USA

## Data Availability

All data contained within this article.
